# Crossing Borders in a Shanghai Kindergarten Through Anji Play: A Cultural Psychology Reflection

**DOI:** 10.1007/s12124-025-09955-y

**Published:** 2026-01-26

**Authors:** Ana Carla Vieira Pio, Huang Xiaoqian, Luca Tateo, Maria Virgínia Machado Dazzani, Pablo Jacinto

**Affiliations:** 1 Student of Interdisciplinary Bachelor Degree in Humanities from Federal University of Bahia, Salvador, Brazil; 2 Student of master’s degree in Early Childhood Education, East China Normal University, Shanghai, China; 3 Theory, Epistemology and Methodology of Qualitative Research, University of Oslo, Oslo, Norway; 4 The Federal University of Bahia. (Universidade Federal da Bahia), CNPq, Brazil; 5 Federal University of Bahia, State University of Southwest Bahia. (Universidade Estadual do Sudoeste da Bahia), Salvador, Brazil

**Keywords:** Boundary crossing, Anji play, Cultural psychology, Early childhood

## Abstract

Based on the assumption that play is crucial to children’s development and education, especially in the early years of the schooling process, the purpose of this article is present and discuss the role of the Anjii Play in Kindergarten in the Chinese context of schooling. We conducted research that sought to describe and analyze how the Anji Play educational model can contribute to the family-school transition and to the crossing of boundaries (symbolic, physical, pedagogical, etc.). Data was collected in a kindergarten in the city of Shanghai, China. Specifically, the research sought to describe both spontaneous and guided school activities; to analyze the planned activities on students’ education; and to examine how educators plan these activities considering the process of boundary crossing, as well as how they evaluate these activities. By using the Anji Play educational method, which promotes children’s enjoyment, engagement and reflection in learning through semi-structured play with natural materials, the kindergarten seeks to implement a way of teaching that emphasizes autonomy and respect for the child. In this sense, the article focuses on the relationship between spontaneous and guided activities using the Anji Play method and their repercussions on the process of boundary crossing for the young students.

## Introduction

According to Souza et al. (2022), play is recognized as a distinctive activity of the early years of life. In this sense, the authors state that play is understood by its ontogenetic adaptive character, as it is a behavior present especially in the immature stages of life, gradually losing its importance over the course of ontogeny (Souza et al., 2022). Thus, play comprises an important strategy both for developing adaptable behaviors in the children’s environment and for developing specific characteristics of this developmental period. Among the benefits that play provides, both to children and young people, are the establishment of bonds of belonging to a social group that shares cultural signs, as well as socialization and identity construction (Kishimoto, 2014 as cited in Souza et al., 2022). Furthermore, Scheu and Xu (2014 as cited in Souza et al., 2022) also postulate that play among adolescents is strongly associated with the enhancement of physical, cognitive, and socio-emotional abilities. Moreover, in the playful context, it is possible that the identity construction process is also strongly linked to the development of linguistic skills. In this sense, the playful repertoire represents a pathway for acquiring skills for future experiences, as well as being a focus of attention and implementation throughout people’s lives (Scheu & Xu, 2014 as cited in Souza et al., 2022).

Following a 15 years long process of revaluation in the international literature (Ahmed et al., 2023), free play has taken on a central role in the teaching-learning process of early childhood, leading Early Childhood Education (ECE) educators in Asia to recognize child-centered and process-oriented play-based pedagogies. These approaches promote active and meaningful engagement, social interaction, and fun learning opportunities for children (Bautista et al., 2023). In this context, there is also a concern with creating spaces to foster this type of play-based learning.

Official school curricula around the world, both in Western and Asian countries, encourage Early Childhood Education (ECE) educators to implement play-based pedagogies. These range from *structured play* (educator-led activities with educational goals) to *free play* (child-led activities that prioritize freedom, choice, and agency). Among the benefits highlighted by Western scholars about this play-based approach are developmental and learning outcomes, including physical, cognitive, academic, socio-emotional, and mental health benefits (Bautista et al., 2023). According to Piaget (1962, cited in Bautista et al., 2023), play reflects stages of a child’s cognitive development, progressing from *functional play* (mastering physical actions), to *constructive play* (using objects to create something new), *symbolic play* (creating scenarios where objects take on new meanings), and finally *games with rules* (structured play involving pre-established rules, typically including competition among participants).

According to Luo et al. (2013, cited in Bautista et al., 2023), the Confucian principle of knowledge (Zhi) directed Chinese parents’ beliefs toward pursuits unrelated to fun and pleasure. Furthermore, the notion of Guan - a Confucian concept referring to training children appropriately according to societal expectations - fosters a top-down educational dynamic (Luo et al., 2013, cited in Bautista et al., 2023). In this model, educators lead the implementation of teaching methods in childcare settings, while children are expected to follow this prescribed approach. Consequently, a pedagogy that positions play as the primary learning tool and centers the child in the educational process is unconventional. As noted, “the ECE curriculum framework in Mainland China can be seen as somewhat revolutionary in its emphasis on the role of play in learning” (Ministry of Education of the People’s Republic of China [MOE-PRC], 2012, cited in Bautista et al., 2023). In contrast to “Guan”, the “Anji Play”, a game model that is entirely led by children and fully empowers them to explore independently, has been promoted in many regions of China and is also a typical curriculum model for children’s autonomous play.

Anji Play is an early childhood education method developed by Ms. Cheng Xueqin for the public early childhood education programs in Anji County, Zhejiang Province, China. In addition to Zhejiang, this method has been adopted by many public programs in several provinces in China, and has become a focus of the Ministry of Education for universal access to public early childhood education in China (Anji Play, n.d.). In her article “Cultivating Young Children’s Positive Emotions: Based on Anji Play,” Cheng (2023) presents the Anji Play educational method as a tool through which children themselves develop ideas, guide and reflect on play (Cheng, 2023). Using natural playgrounds and natural materials for play, the method promotes “children’s self-participation, self-creation, and self-enjoyment during play” (Cheng, 2023, p. 556). In addition, Cheng (2023) explained that play is a fundamental activity for young children and can cultivate brain nerves to produce emotional experiences. Thus, play can effectively promote children’s physical and mental development and inspire educators (Cheng, 2023).

The Anji Play is composed of three major elements: an open game environment, the empowerment of children’s games, and the supportive role of adults. Children could independently explore game methods in an environment free from adult intervention, full of love and a sense of security. Children have ample playtime, no fixed game rules and uncertain playmates. Anji Play seeks to cultivate children’s capacities for engagement and reflection (AnjiPlay, n.d.), its pedagogical framework explicitly advocates for uninterrupted play. This philosophy stems from the foundational belief that children’s autonomous problem-solving - through continuous experimentation with ideas and solutions - is essential for developing lifelong confidence and self-reliance. The approach mandates that ‘during play, educators step back, remain present, and observe with their “hands down, mouths closed, ears, eyes, and heart open” (intervening only when necessary to ensure safety)’ (AnjiPlay, 2024). The role played by educators is merely to reflect on the game experience and share the game feelings with the children through drawing, recording, language and other means after the game ends.

The process in which educators guide children to record and recount their game experiences integrates documentation techniques, utilizing photographs and videos of play episodes as reflective tools. In subsequent review sessions, children articulate their experiences depicted in the media, while educators facilitate the dialogue through carefully crafted, ‘non-leading, open-ended, and clarifying questions designed to reveal children’s thinking processes and play patterns’ (AnjiPlay, n.d.). “Play Stories” is an approach often used when educators guide children to record the play experience, where children first graphically represent their play experiences through drawings, then verbally narrate their depictions to educators for transcription (AnjiPlay, n.d.). This dual-modality exercise serves to deepen reflective practice while honoring children’s expressive agency.

According to Bo Stjerne Tomsen (LEGO Foundation apud Anji Play, n.d.), “Anji Play represents a truly innovative methodology in which carefully selected materials are used for co-construction and reflection, and thereby creating a dialogue of discovery between the child, peers and the educator”. In this sense, since Anji Play is a teaching method centered on play, these activities are not designed and developed in an ordinary or random manner. Each space and tool is prepared in a specific way to serve a particular purpose. Moreover, these activities are child-led. This means children assume a primary and fundamental role in shaping both the activities and their outcomes.

Since boundaries define which elements are foreign and dangerous, familiar elements can provide greater comfort when crossing boundaries. Thus, at the edge, we can find different signs that allow us to move from one zone of meaning to another (Tateo & Marsico, 2021). Through play, the Anji Play approach allows children to cross these boundaries with greater confidence, whether they are transitioning from home to daycare or between classes. Nevertheless, as boundaries shift, children are able to draw upon the repertoire of signs from their prior play experiences. This existing knowledge provides a critical framework that supports them in traversing new challenges.

Starting from the recognition of the importance of play for children in early childhood education, the purpose of the research was to describe and analyze how the Anji Play educational model contributes to the family-school transition and to the crossing of boundaries (symbolic, physical, pedagogical, etc.) for kindergarten students in the city of Shanghai, China. Specifically, the research sought to describe both spontaneous and guided school activities; to describe and analyze the planned activities on students’ education; and to examine how educators plan these activities considering the process of boundary crossing, as well as how they evaluate these activities.

When reviewing the impact of play-based learning according to Asian literature, Bautista et al. (2023, p. 478) identified 7 naturalistic studies, including 5 qualitative ones, which indicate that “play-based pedagogies have the potential to positively impact specific aspects of Asian children’s socio-emotional development”. According to Cheung et al. (2015, as cited in Bautista et al., 2023), in early childhood education that uses play as a foundation for learning, children are more participatory and engaged than those who are academically oriented in preschool. Additionally, it was noted that “children were usually engaged in small-group activities and encouraged to choose their own activities” (Cheung et al., 2015, p. 478 as cited in Bautista et al., 2023). Furthermore, a teacher-directed environment tended to compromise children’s ability to develop their own ideas and relate to classmates and educators (Bautista et al., 2023). When exploring other articles, further benefits were cited, including cognitive and affective outcomes in young children, more opportunities for socio-emotional development, the cultivation of self-reflection and positive self-concept, and reciprocity of prosocial behaviors (Bautista et al., 2023).

Play holds deeper significance for children than superficial observation might suggest. For young learners, play operates as a semiotic system. Semiotics is an activity that constructs and integrates the psyche, responsible for creating a link between the individual and the subjective, collective, and cultural, becoming a constant bond (Valsiner, 2012). In this sense, human experience is a subjectively real, culturally mediated, and constantly recreated reality. Within early childhood education contexts, as children initiate their formal educational trajectory, kindergarten constitutes not merely a transitional threshold but rather a complex landscape of new symbolic systems that children must learn to interpret and negotiate. In this developmental framework, play emerges as a fundamental pedagogical instrument supporting both instruction and knowledge acquisition in early learning environments.

The Anji Play approach exemplifies this principle by employing play as a strategic medium to mediate developmental transitions. This educational methodology extends beyond basic play incorporation, integrating supplementary didactic tools including: (1) narrative-based play documentation (“play stories”), and (2) multimodal activity recording through photographic and video technologies. Through this comprehensive approach, children engage in an iterative learning cycle encompassing: active play participation, verbal description and storytelling, systematic observation, critical reflection, and collaborative discussion of their play experiences.

## Dynamic Transitions

Transitions that occur during the school years were conceptualized by Zittoun (2004, 2007, 2015, 2016) as developmental transitions as a dynamic and dialectical process, marked by periods of reorganization of the systems, the activity structures, and their intersubjective relationships throughout life course. Developmental transitions are defined by a variety of transformations, ruptures, and dynamic neo-formations across various contexts. Zittoun (2015) developed a framework for discussing life trajectories, conceptualizing spheres of experience as social and material configurations, structured by rules and organized through a network of meanings. Zittoun (2007) utilized the concepts of rupture and transition to illustrate the potential for a significant transformation in a system, its surrounding environment, and/or its functioning. This necessitates the implementation of rearrangements to ensure a degree of stability. The ruptures are intransitive changes, critical moments in which an individual’s ongoing adjustment modes are interrupted, potentially resulting from internal or external factors (Zittoun, 2007). They also require individual adjustment or adaptation to the new environment.

Transitions may imply the crossing of boundaries between different contexts (Tateo & Marsico, 2021). Boundaries may be either material or immaterial, physical or imaginary, yet they are the product of an act of signification and interpretation (Tateo & Marsico, 2021). The establishment of boundaries implies the activation of norms within the physical or psychological realm. For instance, setting a boundary between “us” and “them” activates a distinction between elements that can be perceived as foreign and dangerous. Consequently, while a boundary delineates a space in the mind and society, it also establishes an order by specifying what is acceptable and unacceptable (Tateo & Marsico, 2021). Boundaries are responsible for determining the differences between spaces, and different spaces modify the characteristics of people crossing those spaces. Boundaries are marked by specific signs, and signs themselves can become boundaries, as for instance the developmental stages “infancy”, “childhood”, or “adolescence” (Tateo & Marsico, 2021). The transition from one stage of life course to the other is marked by changes in both the persona and the context.

### Boundaries in Education

One of the first boundaries that people usually cross is the one between infancy in family and formal education (Marsico et al., 2013). For instance, the beginning of kindergarten also serves to demarcate a new environment replete with signs and symbols that are unfamiliar to the children. Such an institutionalized transition, requires actions of preparation and scaffolding for the child. According to the principles and practices of Anji Play, a teaching method that promotes children’s leadership and autonomy through play in their learning process, “children are trusted to resolve their own conflicts and overcome most challenges” (Anji Play, n.d.). Cheng (2023) presented the Anji Play educational method as a tool for children to develop their own idea generation, management, and reflection through play, skills that are beneficial to children’s school development.

In the Anji Play method, children lead their own play, which means they bring their unique perspectives and experiences into the classroom. When they face a new challenge, they don’t start from scratch—they use what they’ve learned before to find a solution. This bank of prior knowledge acts as a personal guidebook, giving them the confidence and comfort to venture into the unknown and explore new possibilities.

Play can be utilized as an instrument for facilitating these transitions, particularly in the context of child development and learning. The child is enabled to experiment with autonomy in the process of crossing boundaries. Subsequently, this will affect the way in which the child navigates the crossing and its timing. Furthermore, given that play is a familiar activity, it can facilitate the crossing process. In this regard, Anji Play appears to be a potentially valuable tool for assisting children in navigating this transition, so it has been adopted as a frame of reference for the present study.

## Method

### The Research Context

The study was conducted in a kindergarten in Shanghai, one of the first Chinese seaports to open to Western trade and now one of the largest seaports in the world and a major industrial and commercial center in China. The Anji Play educational method “is practiced in public early childhood programs in all 34 provinces and administrative regions of China” (Anji Play, n.d.) but also in the urban area of Shanghai.

Considering “the child’s need for extensive, uninterrupted, and unguided real play, play that is initiated, directed, and determined by the child” (Anji Play, n.d.), the Anji Play teaching method promotes spontaneous, child-centered activities that encourage reflection. It is also sensitive to the environmental dimension, sustainability and care for nature (Li et al., 2019). The kindergartens that follow this structure are characterized not only by minimally structured and open environments with different areas, without a defined theme or topic (Anji Play, n.d.), but also by professionals who are guided by an understanding of these principles (Anji Play, n.d.). To meet the activity criteria, the kindergarten visit featured a large, well-equipped environment. The school building consisted of multiple classrooms, a cafeteria, administrative offices, an infirmary, and other standard facilities. Specifically, each classroom was thoughtfully arranged to support early childhood development, featuring walls decorated with the children’s artwork and activity photographs. The space included a designated reading area stocked with a variety of children’s books, storage containers filled with building blocks, and child-sized tables and chairs for group activities. Additionally, the classroom was equipped with a projector for multimedia learning, an attached bathroom for convenience, and a separate sleeping room furnished with multiple bunk beds. Meanwhile, the outdoor play area—located separately from the main building—was designed to encourage active exploration, offering swings, slides, climbing structures, barrels, painting supplies, gardening tools, sandboxes, bicycles, and a dedicated planting area, all set among trees and greenery that enhanced the natural learning environment.

## Spontaneous Activities

**Fig. 1:**
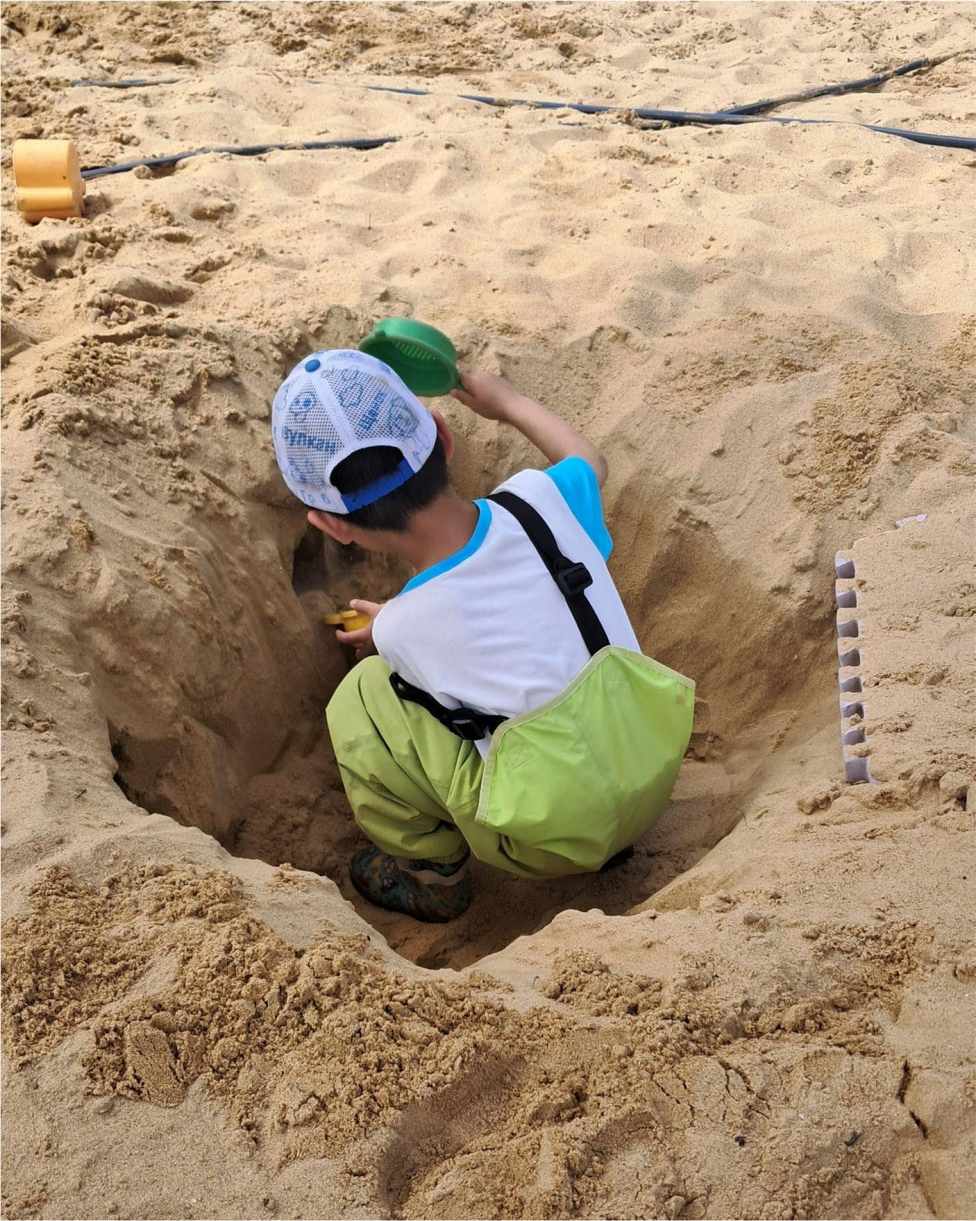
Child playing in the sandbox. Source: Pio, A. (PHOTOGRAPHER). 2024

Spontaneous activities, as the name suggests, are those in which the children have more autonomy to choose what to do and how to do it, using the school playground as their environment. Of course, spontaneity doesn’t mean that the situation is constrainless. The school environment is filled with signs, materialities, temporalities that frame children-initiated actions (Fig. 1). So, in the context of this study, spontaneous activities refer to those playful moments mainly initiated and organized by the children independently within the framework set by the adults.

According to the Anji Play model (n.d.), those materials encourage children to explore cause and effect relationships, collaborate with peers, imagine, create, and challenge themselves at their highest level of ability. In addition, as a space organized by materials rather than by theme or topic, the purpose of outdoor activities is to allow the child to explore, imagine, and create (Anji Play, n.d.). However, at least in the school where the data were collected, each class spends a month in each area. Thus, the children rotate through different activities and instruments over time.

In the rotation system described above, the groups of children are allocated to predetermined spaces for spontaneous activities, reflecting the institution’s curricular intent to maintain a degree of control over developmental opportunities. This rotation also facilitates intra-institutional transitions, as the spaces designated for children’s spontaneous activities present diverse challenges and opportunities for skill-building and interaction with the environment and one another.

## Guided Activities

**Fig. 2:**
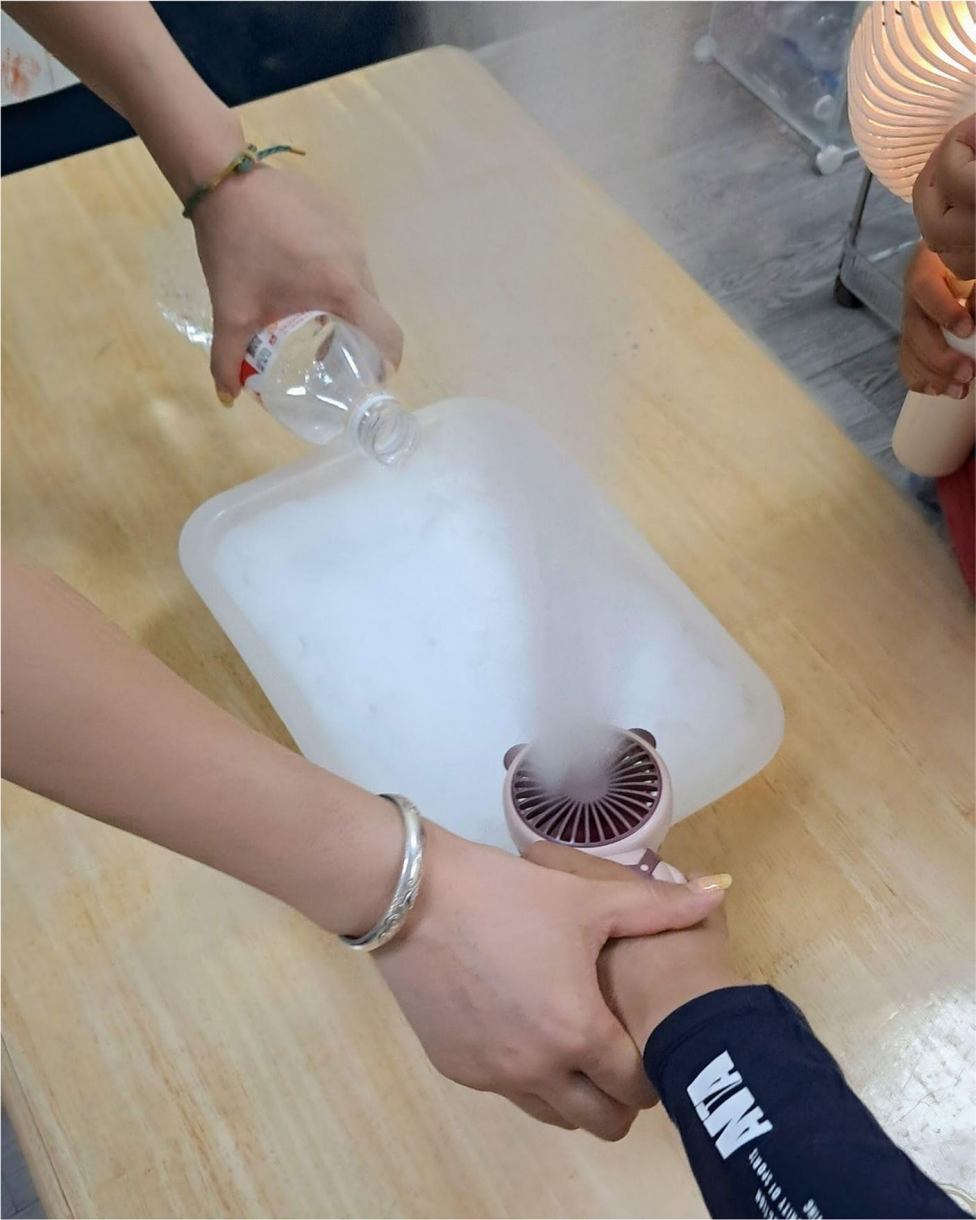
The educator leads the child in experimenting. Source: Pio, A. (PHOTOGRAPHER). 2024

Planned activities, on the other hand, are those prepared in advance by the kindergarten staff with a predefined objective and more or less planned sequence. Unlike spontaneous activities, children are guided by educators in these activities (Fig. 2).

Planned activities are not totally rigid. Children have their own time to do what they want within the spaces and with the tools provided for the activity, and they also have time to engage in the educational activities developed by the kindergarten’s staff.

Although the activities are guided, they are still in line with the Anji Play purpose of promoting children’s autonomy. Therefore, while the activities are developed by the school, the children still play a central role in their learning. For example, in the classroom observed, the routine guided activities were characterized by drawing, storytelling, discussion of previously free activities, and science experiments. Thus, the children were responsible for narrating their play, discussing the story told and the previously completed activity, and conducting scientific experiments with the educators.

In this sense, the activities still prioritize placing the child in an active position within the scenario. But not only the scenario, the *true play*, the method that Anji Play (n.d.) defends, has the purpose of bringing engagement to learning. In guided activities, however, the children have to follow the instructions of their educators.

### Participants

The class involved in the study hosted children between 5 and 6 years old. The class was selected after a period of observation because as a group of elderly children have longer experience of kindergarten life. The present study focused on the perspective of educators, as they manage activities and have the opportunity to observe different classes of the same age.

Recruiting educators was difficult because the field work took place when the holiday break was approaching. Thus, many educators were busy with the final activities of the kindergarten as well as preparing the school and educational plan for the next semester. Just three educators with the profile required by the research design volunteered to participate in the research. The participant selection process was designed to ensure qualified representation of classroom perspectives. Educators were required to: (1) hold primary instructional responsibility as the lead teacher of their assigned class, ensuring first-hand knowledge of daily pedagogical practices; and (2) demonstrate availability for semi-structured interviews, conducted either via secure video-conferencing platforms or on-site to accommodate scheduling preferences. These criteria guaranteed that participants possessed both the operational authority to discuss the practice of Anji Play and the flexibility to engage meaningfully with the research thematic.

## Research Design

This qualitative and descriptive research was developed through empirical procedures that included field observations in a kindergarten located in Shanghai, China, followed by semi-structured online and face-to-face interviews with three educators. These interviews explored emergent themes in both planned and spontaneous classroom and outdoor activities. The observations were conducted in a participant configuration and lasted for two months, in 2024, with a frequency of three times per week. The instruments used are described below.

## Instruments

The first two authors (A.A. and B.B.) gathered data using two primary methods: participant observation, which included taking photographs and field notes; and semi-structured interviews. A kindergarten visit schedule was structured in three visits per week, for three hours from 9:00 to 11:30, on Monday, Wednesday, and Friday, during May to June, during the year of 2024. The days of the visits were decided based on the availability of the students and educators. To facilitate communication during observational visits, translation was provided by Chinese university students for interactions between the Brazilian students and the Chinese educators, school directors, and peers.

In addition, three semi-structured interviews were conducted with the participating educators. Semi-structured interviews are a widely used qualitative research method that combines pre-established questions with the flexibility to explore open-ended responses. They are particularly valuable for gaining in-depth insights into participants’ thoughts, beliefs, experiences, and motivations, while allowing the researcher to probe new or unexpected topics. This approach is well-suited for exploring complex issues, as it balances structure with adaptability (Mashuri et al., 2022). The questions addressed (1) a description of spontaneous and guided activities, (2) how these activities can support transitions from home to school and from preschool to elementary school and (3) how these activities would be assessed. The interviews were recorded via a mobile device. Also, since one of the researchers tasked with structuring and conducting the interview was Chinese, it was also possible to perform a translation from English to Chinese if any participants had doubts. Later, the interviews were transcribed with the aid of artificial intelligence for analysis.

### Data Analysis

The data analysis was primarily conducted by the first author, in constant dialogue with the other authors, aiming to establish a shared understanding of the interpretation of the research data. Describing the observations made during the researcher’s visits to the field was essential for structuring the analysis and generating insights related to the research topic. These observations were analyzed through the compilation and organization of field notes and photographs. The goal was to identify, based on what was documented during the visits to the kindergarten, which situations represented the core elements of the study: planned or spontaneous activities and the developmental transitions between them. During the data analysis process, the field notes and photographs were gathered and categorized to better understand the relationships children established with both planned and spontaneous activities, as well as how they navigated the transitions between these types of engagement.

The results of the observation analysis were triangulated with the thematic analysis of the interview data (Clarke & Braum, 2017). The same categories developed during the observation phase were used in this triangulation, and excerpts from the interviews that corresponded to these categories were selected to explore their meanings in relation to the research questions. Following the principles outlined by Gil (2008), these categories were refined to more accurately represent the researched reality, based on the researchers’ interpretations and grounded in the study’s theoretical framework. Thematic analysis, through a process of coding, enabled a more detailed understanding of the interview data. The resulting categories were described and contrasted with the theoretical foundations concerning developmental transition processes (Tateo & Marsico, 2021; Zittoun, 2007, 2015). Considering the child’s transition into kindergarten and the different atmospheres involved, the theoretical concept of boundary crossing, borrowed from Cultural Psychology, was chosen as the primary lens for data analysis, as it offers valuable tools to explore and support this theme.

## Results

The results are structured around key elements that contribute to the understanding of transitional processes experienced by children in a kindergarten setting, particularly in the context of child-led and teacher-oriented activities. Afterwards, we explore play as a sign and reflect on its meaning for the educators participating in the study. Finally, we examine how these processes relate to the crossing of borders and developmental transitions (Zittoun, 2007; Tateo & Marsico, 2021) of children in the kindergarten environment.

### Play as a Sign

*True play* is so called because it promotes “deep and uninterrupted engagement in the activity of one’s choice” (Anji Play, n.d.). It can be characterized by “observable experiences of risk, joy, and deep engagement” (Anji Play, n.d.). According to the Anji Play (n.d.) method, *true play* can create “ecologies that prioritize the understanding of learning and development in their respective communities”. The same perspective as in the Anji play is brought by the Teacher interviewed.

When asked about the dynamics of spontaneous activities, activities that have the same characteristics as *true play*, Teacher I emphasizes:*“For them*,* the self-publishing activities have more initiative. They can use their own initiative to participate and choose. Yes*,* I think when the child has the initiative*,* he can have more experiences*,* learn*,* and interact with his peers.”* In this sense, Teacher I recognizes in spontaneous activities an opportunity for the child to not only put their decision-making ability into practice, but also to train new skills and strengthen bonds among peers.

### Spontaneous and Guided Activities Capable of Facilitating Boundary Crossings

When asked about the characteristics and purposes of guided activities, both Teacher I and Teacher II raised points that differ but still complement each other.

Teacher I states that:

- “We can talk through these materials. It’s like a bridge because children they didn’t have enough ability to talk directly without any help.” She emphasizes the use of materials, both instrumental and the activities themselves, as an important tool capable of helping the child learn certain skills or facilitating this process.

The teacher II observes that *“All activities are conducted with respect for the children as the foundation. Each requires teachers to observe*,* understand each child’s situation*,* and provide support accordingly.”* Although these activities prioritize the child’s leading role in their own learning process, Teacher II emphasizes that the teachers remain present and attentive during the activities, offering a different interpretation if necessary.

In this way, spontaneous and guided activities provide—one more than the other—for the child to lead more autonomously in determining the paths for learning. This autonomy aims to respect the uniqueness of each child by allowing them to make their own decisions about how these activities will be managed. However, those children who, for some reason, have more difficulty guiding or managing an activity will receive more assistance from certain materials or from the teachers.

### Autonomy in Which Boundaries Can Be Crossed

During the interview, Teacher I also emphasizes spontaneous activities without adult interference as an important factor in the child’s performance in the kindergarten:

- “Because I discovered when I interrupt their play, they will change or influenced by me […] is children’s play, not adults or not teachers play […] They should learn how to start a play by themselves, how to solve the problem during the plays. If teacher will interrupt or help them always, their opportunities of their learning might be of learning by themselves.” (Educator I).

In this explanation, Educator I emphasizes the importance of children’s autonomy in making their own decisions as a method for them to learn to solve potential problems without necessarily relying on an adult. In this way, Teacher I emphasizes the importance of the child leading the activity as a way to also exercise their capacity for planning, decision-making, and problem-solving. This also contributes to the child exercising autonomy in defining what their problems are and how they will be managed. In this sense, before solving a problem, the child practices the activity of identifying the potential problem.

### How their Transitions Are Evaluated

When asked about how each child could be evaluated, Teacher II notes that evaluations are conducted with a focus on the child and their individuality: *“Analyze each child’s characteristics*,* as their paintings are linked to their thoughts*,* and each child is different.”* Following the purpose of the activities, the assessment of the children also follows each one’s individuality. Thus, there would not be a single parameter that measures every child, but rather an assessment of how each one has developed within their own possibilities.

In this way, even though guided activities involve teacher supervision and are structured around regular activities, the way the performance of these children is analyzed is not focused on who is right or wrong, but rather on how they handle new scenarios.

### Anji Play as a Guide for the Future

Furthermore, the Anji Play teaching method can also serve as a guide for the future. Teacher I states that: “*So in the primary school*,* teacher will… maybe in the teacher’s eyes*,* he is not that clever. But in his place* (the kindergarden), *he knows he is clever. He will be… like… confident… hum… know more about him self-conscious will be more.*” In this sense, despite a change in how the dynamics will be organized, Teacher I states that the child will be able to access the emotional skills that were trained during kindergarten activities and face future challenges.

## Discussion

In their work, for example, Meng and Heuschkel (2020) used drawing as a tool to discuss features of children’s drawings, family activities, and parenting styles. Since children do not have the ability to express their needs, motivations, and family about their family (2020), the activity of drawing was chosen as a tool to understand their perspective. In this case of a particular child-directed activity in the kindergarten, the drawing activity is part of the Anji Play curriculum practice called ‘Playstory’. This is a form of narrative drawing in which the children record the process, action, outcome, emotions and other independent experiences of the play, after the children have played independently and spontaneously in their previous activity. However, although the children were free to construct their drawings in terms of composition, colors and other aspects, the theme remained limited to a previous activity carried out in a specific kindergarten environment, and the educators corrected the children whenever their work deviated from the suggested guidelines. So there was a degree of freedom, but with an implicit idea of what they should draw.

And because it is a child-directed activity, this form of play “stimulates their internal motivation to learn” (Cheng, 2023). In this sense, it brings the importance of these activities as a tool for children to develop their autonomy. In addition, the motivation to learn can be explained by the meaning that play carries. As a familiar sign, play can carry extraordinary meanings, from something familiar to something with a unique and individual history.

Considering that signs provide a space for dialogue and discussion as well as potential confusion (Tateo & Marsico, 2021), when the kindergarten allows children to use their own meanings to guide their learning tasks, it also uses this familiar sign to help children navigate different boundaries. In this sense, through this connection, play can be an important sign in the transition from one boundary to another.

As previously established, a child’s entry into kindergarten represents a rupture from their familiar organizational systems, and play can serve as a crucial tool for fostering adaptable behaviors (Souza et al., 2022) during this transition. Consequently, a period of transition (Zittoun, 2007) necessitates adjustment periods. However, this is not the only factor to consider. In an effort to establish new arrangements that promote stability, as long as signs provide a space for dialog and discussion (Tateo & Marsico, 2021), the use of already-known signs can facilitate this activity. Thus, the use of play at this stage of boundary crossing also uses a sign that is already familiar and internalized to engage with other, unfamiliar signs.

Since signs can have a regulatory function (Tateo & Marsico, 2021), play, as a pedagogical tool that allows children to choose the activities in which they engage, can take on this regulatory function and help children navigate the different boundaries they encounter in kindergarten and beyond. In other words, kindergarten is surrounded by signs and meanings that are unfamiliar to children. Learning these new signs can be facilitated by bringing something the child already knows into contact with unfamiliar signs. Because children have the opportunity to choose which activities to do, they can also engage in activities that have special meaning to them. In this way, through play, children can reconstruct existing signs and also create new boundaries, approaching behaviors and norms that were previously seen as outside and interpreting and internalizing them as inside.

However, according to Zittoun (2007), new contexts emerge around new forms of organization. For this reason, children may have difficulty moving from one boundary to another because, according to Educator I, some children may need more support from educators than others. In this sense, spontaneous activities may not always have the same effect on all children.

As mentioned earlier, the guided activities aim to combine two elements: the familiar and the unfamiliar. To achieve this, guided activities are planned to include areas that children may not be able to reach on their own. However, even though the activities are guided, the priority remains to promote the child’s agency in learning. In this way, educators work together with the children so that they play an active role throughout the process.

Thus, it can be observed that both spontaneous and guided activities serve the purpose of connecting the child from one boundary to another, facilitating the transition between different spaces and their signs.

Besides, since the transition process requires moments of reorganization, as mentioned for Zittoun (2004, 2007, 2015, 2016), when a child has the autonomy to lead activities, it can help boundary crossing because the child can choose which boundaries to cross.

On the other hand, encouraging this autonomy can also influence how a child deals with crossing new boundaries and which boundaries are crossed. This is because interrupting a child’s activity by presenting a solution to a potential conflict also means presenting signs and their meanings, which may not be the same thing to the child. Still following the ideas of Zittoun (2007) that critical moments require processes of adjustment, facing new signs can become a challenge. In addition, signs serve as a guide for experiencing phenomena and environments (Tateo & Marsico, 2021). In this way, the familiar signs can provide the characteristics that the Anji Play method defends as the most important during the play.

Thus, when children have the opportunity to explore possible ways to solve problems, whether in free or guided activities, they are also entering boundaries independently, through their own signs. Therefore, what the kindergarten evaluates is not which boundary is crossed, but whether and how the child can cross the boundary.

Understanding that the Anji Play method offers children an alternative way to develop certain skills, such as problem solving, guided activities are evaluated without losing sight of the purpose of spontaneous activities.

In addition, the action of transition requires the individual to negotiate certain adjustment or adaptation strategies with the environment (Zittoun, 2015) according to personal differences. Based on this, it is not the purpose of this kindergarten to examine the transition without taking any consideration of the individual characteristics of each child. Thus, the teacher pays attention to and is very interested in each person’s unique way of crossing the boundaries and whether they are able to negotiate this transition. In this sense, even though regular school may not offer the same activities and goals, children can still use the skills developed through spontaneous and guided activities when they leave kindergarten.

Therefore, the Anji Play teaching method also considers how children can use these skills in the future. Since developmental transitions are responsible for creating new dynamics (Zittoun, 2007), by marking a boundary, Anji Play also provides a set of signs responsible for guiding children along their lives. In this sense, boundaries are not only external; they are always present and guide the individual’s actions throughout life (Tateo & Marsico, 2021).

In this way, the physical transition from kindergarten to regular school allows the child to retain the skills developed through play. In this way, the skills developed by the kindergarten can become a mental boundary and take on the responsibility of regulating the child in this new environment, potentially providing the same benefits as during the future years in a regular school.

## Conclusion

Our analysis showed how different boundaries can determine how the dynamics of an environment relate to the way a child will deal with the new atmosphere of the school. Play as a pedagogical tool can represent a regulatory function for the child. By acting as a familiar cultural reference, play facilitates the negotiation of this new setting and fosters the adaptive behaviors necessary for a successful transition.

In addition, play can provide the child with feelings such as “self-participation, self-creation, and self-enjoyment” (Cheng, 2023) that are beneficial to physical and mental development. In early childhood education, these skills can facilitate boundary crossing in such a way that the child is better equipped to handle new signs with autonomy and confidence. Thus, it can be observed that Anji Play activities are designed in such a way that the child does not feel overwhelmed by this boundary crossing.

Given that the Anji Play method cultivates self-confidence and autonomy in children, empowering them to play and solve problems, future research should examine how children adapt these competencies to employ analogous strategies when crossing boundaries between educational stages, and it can serve as a guide for the future.

Considering that the research sought to investigate the class of children aged 5–6, it might be interesting for future research to also observe younger classes. Since the observation began in the middle of the first semester, it was not possible to observe the initial contact of the younger children with the kindergarten.

## Data Availability

The data are provided within the manuscript.
